# Primary Palatine Tonsil Non-Hodgkin Lymphoma in Western Romania: A Comparison of Lower-Stage and Advanced-Stage Disease

**DOI:** 10.3390/hematolrep17020017

**Published:** 2025-03-28

**Authors:** Raluca Morar, Norberth-Istvan Varga, Delia Ioana Horhat, Ion Cristian Mot, Nicolae Constantin Balica, Alina-Andree Tischer, Monica Susan, Razvan Susan, Diana Luisa Lighezan, Rodica Anamaria Negrean

**Affiliations:** 1 Doctoral School, Department of General Medicine, “Victor Babes” University of Medicine and Pharmacy, Eftimie Murgu Square No. 2, 300041 Timisoara, Romania; raluca.morar@umft.ro (R.M.); norberth.varga@umft.ro (N.-I.V.); 2 ENT Department, “Victor Babes” University of Medicine and Pharmacy, Eftimie Murgu Square No. 2, 300041 Timisoara, Romania; horhat.ioana@umft.ro (D.I.H.); balica@umft.ro (N.C.B.); tischer.alina@umft.ro (A.-A.T.); 3 Department of Internal Medicine I, Centre for Preventive Medicine, “Victor Babes” University of Medicine and Pharmacy, Eftimie Murgu Square No. 2, 300041 Timisoara, Romania; susan.monica@umft.ro; 4 Department of Family Medicine, Centre for Preventive Medicine, “Victor Babes” University of Medicine and Pharmacy, Eftimie Murgu Square No. 2, 300041 Timisoara, Romania; razvansusan@umft.ro; 5 Multidisciplinary Research Center for Malignant Hematological Diseases, “Victor Babes” University of Medicine and Pharmacy, Eftimie Murgu Square No. 2, 300041 Timisoara, Romania; lighezan.diana@umft.ro; 6 Department of Preclinical Disciplines, Faculty of Medicine and Pharmacy, University of Oradea, 410073 Oradea, Romania; rodicanegrean@uoradea.ro

**Keywords:** palatine tonsil, tonsillar lymphoma, Non-Hodgkin lymphoma, NHL, head and neck lymphoma, ENT region lymphoma

## Abstract

**Background**: Limited data exist on primary palatine tonsil Non-Hodgkin lymphoma (NHL) from regions with constrained healthcare access. This study investigated this malignancy in Western and South-Western Romania, comparing lower-stage (Ann-Arbor I-III) and advanced-stage (IV) disease. **Methods**: A retrospective cohort study (2010–2019) at a tertiary referral hospital included 59 patients with primary palatine tonsil NHL. Data on demographics, clinical presentation, comorbidities (including viral hepatitis B/C), histology, International Prognostic Index (IPI) score, treatment, and outcomes were collected. Statistical comparisons between lower-stage (*n* = 26) and advanced-stage (*n* = 33) groups were performed. **Results**: A high proportion presented with advanced-stage disease (55.9%). The advanced-stage group had significantly more B symptoms (90.9% vs. 69.2%, *p* = 0.038) and elevated LDH levels (93.9% vs. 57.7%, *p* = 0.013). Viral hepatitis B and/or C infection was more frequent in advanced-stage disease (30.3% vs. 15.4%, *p* = 0.44). Combined chemoradiotherapy was more commonly used in lower-stage disease (38.46% vs. 12.12%, *p* = 0.019). There was no statistically significant difference in relapse rates between the groups. **Conclusions**: This study highlights the substantial burden of advanced-stage primary palatine tonsil NHL in Western Romania, suggesting a need for improved early detection. The association between viral hepatitis and advanced-stage, although not statistically significant, warrants further investigation. These findings may inform tailored management approaches in resource-constrained settings.

## 1. Introduction

Lymphomas are a diverse group of hematologic malignancies originating from lymphocytes, the white blood cells that are integral to the body’s immune system [1,2]. They are broadly categorized into Hodgkin and non-Hodgkin lymphomas (NHL), with the latter encompassing a wide array of subtypes distinguished by their unique histological and clinical characteristics. In the head and neck region, NHL can present in both nodal and extranodal sites, including the oral cavity, although such occurrences are relatively rare [3,4,5,6]. Typical manifestations in this area include swelling, pain, and ulceration [7,8]. Primary Non-Hodgkin lymphoma of the palatine tonsil is a rare clinical entity, often presenting a diagnostic challenge due to its insidious onset and mimicry of benign inflammatory conditions [8,9,10]. This diagnostic ambiguity frequently leads to delays in appropriate treatment, particularly in regions with limited access to specialized otolaryngologic and oncologic services. While the palatine tonsils, as the largest lymphoid aggregates within Waldeyer’s ring, are the most common site of extranodal NHL in the head and neck, comprehensive data on this specific malignancy remain scarce, particularly from areas with poor access to healthcare settings.

The existing literature, primarily based on data from well-funded and well-developed healthcare systems, identifies diffuse large B-cell lymphoma (DLBCL) as the most prevalent histological subtype, with 5-year survival rates varying significantly depending on disease stage and treatment approach [7,8,9,10,11,12,13,14]. The International Prognostic Index (IPI), incorporating factors like age, stage, LDH levels, and performance status, is a well-validated tool for predicting outcomes in NHL [13,14]. However, the applicability of these findings to populations with different socioeconomic backgrounds, healthcare access patterns, and potential co-factor exposures remains uncertain. Specifically, in regions like Western and South-Western Romania, where a significant proportion of the population resides in rural areas with limited access to specialized medical care, the interplay between delayed diagnosis, advanced-stage presentation, and co-morbidities like viral hepatitis B and C requires careful investigation. These viral infections, known to be associated with an increased risk of certain NHL subtypes, may further complicate the clinical course and treatment response in patients with palatine tonsil NHL.

To address this critical knowledge gap, we conducted a retrospective study at a major tertiary referral hospital in Western Romania, analyzing a decade of data on patients diagnosed with primary palatine tonsil NHL to explore how rural healthcare access disparities—rather than resource constraints at treatment centers—might shape clinical presentation and outcomes in this region. This study aimed to characterize the clinical presentation, treatment patterns, and outcomes of this malignancy in a region with certain healthcare challenges.

## 2. Materials and Methods

### 2.1. Study Design and Population

This hospital-based observational, retrospective cohort study was conducted at the ENT (Ears, Nose, and Throat) ward of the Timisoara Emergency Clinical Hospital, a major tertiary referral center serving a large population in the Western and South-Western regions of Romania. Data were collected retrospectively in 2024. The study population included adult patients (aged 18 or older) diagnosed with primary palatine tonsil Non-Hodgkin lymphoma (NHL) over a ten-year period, from 2010 to 2019. Patients were divided into two groups based on their Ann-Arbor stage at initial presentation: a lower-stage group (Ann-Arbor stages I, II, and III) and an advanced-stage (or late-stage) group (Ann-Arbor stage IV). The follow-up period started upon treatment completion and included regular check-ups at 6, 12, 24, 36, and 60 months post-treatment. Patients were eligible for inclusion if they had a confirmed diagnosis of primary palatine tonsil NHL based on histopathological examination. Cases were classified as ‘primary palatine tonsil NHL’ when the palatine tonsil was the site of initial presentation or diagnostic biopsy, without evidence of other structures of Waldeyer’s ring being involved. Exclusion criteria were: minors (age below 18) and patients with incomplete follow-up data (less than 60 months). Patients who died during the 60-month follow-up period were included in the study, and their data up to the point of death were analyzed.

### 2.2. Data Collection

For each included patient, we recorded a comprehensive set of data, encompassing demographic characteristics (age, sex), area of residence, and clinical features. Disease staging followed the Ann-Arbor classification system, which assesses lymphoma based on disease spread. A standardized set of procedures was conducted upon admission for severity assessment. These procedures included: (1) comprehensive ENT evaluation with clinical examination and nasopharyngeal fibroscopy; (2) biopsy or surgical excision of the palatine tonsil, followed by histopathological and immunohistochemical analysis; (3) laboratory tests, including complete blood count, lactate dehydrogenase (LDH) as a marker of tumor aggressiveness, and serological screening for human immunodeficiency virus (HIV), hepatic B virus (HBV), and hepatic C virus (HCV); (4) imaging via cervical–thoracic–abdominal–pelvic computer cosmography (CT) scans for all patients, with PET-CT added for limited-stage cases (I-III) when available; and (5) bone marrow biopsy to assess marrow involvement.

The presence or absence of B symptoms, which are systemic symptoms indicative of more aggressive disease and include fever, night sweats, and unexplained weight loss of more than 10% of body weight in the preceding six months, was also documented. Comorbidities were systematically recorded and grouped into categories such as cardiovascular diseases, diabetes, obesity, previous history of malignancy, thyroid gland or other endocrinological conditions, stroke, psychiatric disorders, infectious diseases, smoking, alcohol consumption, etc. The histological subtype of lymphoma was determined based on pathological examination.

The Eastern Cooperative Oncology Group (ECOG) performance status was used to evaluate the functional status of patients at the time of diagnosis. The International Prognostic Index (IPI) was used to assess the prognosis of patients at the time of diagnosis. The IPI is a clinical tool that predicts the outcome for patients with aggressive non-Hodgkin lymphoma based on five factors: age (≤60 years vs. >60 years), serum lactate dehydrogenase (LDH) level (normal vs. elevated), Eastern Cooperative Oncology Group (ECOG) performance status (0–1 vs. 2–4), Ann-Arbor stage (I-II vs. III-IV), and number of extranodal sites of disease (≤1 vs. >1). Each factor present adds one point to the score, stratifying patients into four risk groups: low (0–1), low-intermediate (2), high-intermediate (3), and high (4–5).

Data on the type of treatment received, including chemotherapy, radiotherapy, and surgical excision of the palatine tonsil, were collected. The primary outcomes of interest during the 60-month follow-up period were relapse, defined as the reappearance of lymphoma after a period of remission; death from any cause; and progression-free survival (PFS), defined as the absence of detectable disease with no relapse during the entire follow-up period.

### 2.3. Ethical Considerations

This study followed the principles of the Helsinki Declaration on Medical Protocol and Ethics. Ethical approval was obtained from the Ethics Committee of the “Victor Babes” University of Medicine and Pharmacy, Timisoara, and the Timisoara Emergency Clinical Hospital (Reference number 52 of 11 December 2019, rev E-489 of 7 February 2025). Patients provided general consent for data use upon hospital admission, as per general clinical practice.

### 2.4. Statistical Analysis

Descriptive statistics were used to characterize the lower-stage and advanced-stage groups. Continuous variables were summarized as means and standard deviations (SD), and categorical variables were summarized as frequencies and percentages. To determine the distribution of continuous variables, the Shapiro–Wilk test was applied, and histograms were visually inspected. The Mann–Whitney U test was performed for non-normally distributed continuous variables between groups. Fisher’s exact test was used to compare the following categorical variables: sex, area of residence, presence of B symptoms, elevated LDH levels, histological subtype, and treatment received. For the International Prognostic Index (IPI) scores, patients were grouped into two categories (High-Intermediate + High vs. Low + Low-Intermediate), and Fisher’s exact test was used to compare the distribution between the limited and advanced stage groups. Among surviving patients, the rate of relapse was compared between groups using Fisher’s exact test, and a multivariable logistic regression analysis was performed, to assess factors associated with relapse while adjusting for confounders. A *p*-value of less than 0.05 was considered statistically significant for all analyses. All statistical analyses were performed using SPSS version 27.

## 3. Results

### 3.1. Description of Study Population

This study included 59 patients diagnosed with primary palatine tonsil NHL, divided into a lower-stage group (Ann-Arbor stages I-III, *n* = 26) and an advanced-stage group (Ann-Arbor stage IV, *n* = 33). In the lower-stage group, two patients were diagnosed with stage I, nine patients with stage II, and 15 with stage III disease.

The mean age of the lower-stage group was 61.34 years (SD = 10.51), while the advanced-stage group had a slightly higher mean age of 64.85 years (SD = 13.36). The sex distribution was relatively balanced in both groups, with a slight female predominance. Area of residence differed slightly between groups, with a larger proportion of patients in the advanced-stage group residing in rural areas (63.63%) compared to the lower-stage group (50%).

The presence of B symptoms was considerably more frequent in the advanced-stage group (90.90%) than in the lower-stage group (69.23%). Similarly, elevated initial LDH levels were far more prevalent in the advanced-stage group (93.93%) compared to the lower-stage group (57.69%). Regarding comorbidities, both groups exhibited a high prevalence of cardiovascular disease, obesity, alcohol consumption, and diabetes. Notably, viral hepatitis B and/or C infection was present in 30.30% of the advanced-stage group, compared to 15.38% of the lower-stage group. Specifically, three patients from the lower-stage group and six from the advanced-stage group had chronic hepatitis B. The distribution of histological subtypes was similar between the two groups, with Diffuse Large B-cell lymphoma (DLBCL) being the predominant subtype in both the lower-stage (76.92%) and advanced-stage (78.78%) groups. The International Prognostic Index (IPI) scores differed between the groups. The advanced-stage group had a greater proportion of patients with high-intermediate and high IPI scores (45.45% and 24.24%, respectively) compared to the lower-stage group (38.46% and 11.53%, respectively). The majority of patients in both groups received chemotherapy as part of their treatment, with R-CHOP (Rituximab, Cyclophosphamide, Doxorubicin, Vincristine, and Prednisone or Prednisolone) being the most common regimen. Patients with the follicular subtype from both groups received the Obinutuzumab-CHOP regimen. A key difference in treatment was the higher proportion of patients in the lower-stage group receiving combined radiotherapy and chemotherapy (38.46%) compared to the advanced-stage group (12.12%). Radiotherapy in the late-stage group was utilized only for palliative purposes. Total surgical excision was performed in five cases on patients with lower-stage disease.

In terms of outcomes, four patients from the late-stage group, and one from the lower-stage group, died during the follow-up period. Their data was analyzed until the point of death. Of the 25 surviving patients in the lower-stage group, seven experienced relapses at various times during their 60-month follow-up period, while 18 patients experienced complete remission. In the advanced-stage group, out of the 29 surviving patients, 13 relapsed during follow-up, and 16 experienced progression-free survival (PFS). At the time of the last check-up, however, all surviving patients were considered to be in remission, with no detectable disease at the full-body CT scan.

The overall summary of the study population is presented in Table 1.

### 3.2. Characteristics of Non-Survivors During Follow-Up

Four patients with stage IV (late-stage group) and one with stage IIIA (lower-stage group) died during the 60-month follow-up period. At diagnosis, the non-survivors had a mean age of 70.2 years (SD = 6.6), which was higher than the overall cohort’s mean age. All five were female and lived in rural areas. All five patients presented with B symptoms and had elevated LDH levels at diagnosis. Each of them had multiple comorbidities, including cardiovascular disease (all had hypertension and NYHA class II or III heart failure; one had atrial fibrillation, and two had a history of myocardial infarction) and obesity. Additionally, two had diabetes, two reported a history of smoking, and two had a prior history of stroke and mixed dementia. One patient had previously undergone a thyroidectomy for Graves’ disease. Histologically, all cases were classified as diffuse large B-cell lymphoma. One patient from the late-stage disease group refused all treatment, due to personal reasons, and the remaining four received the R-CHOP chemotherapy regimen. The patient who refused treatment had a ‘Low-Intermediate’ IPI score, but the other four patients had a ‘High’ IPI score. Death occurred in the period of time between 12 and 60 months after diagnosis in all five cases.

### 3.3. Comparison of Lower-Stage and Advanced-Stage Groups

The mean age of patients in the advanced-stage group (64.85 years) was slightly higher than that of the lower-stage group (61.34 years). This difference was not statistically significant (*p* = 0.310). The distribution of sexes did not differ significantly between the lower-stage and advanced-stage groups (*p* = 0.519). A greater proportion of patients in the advanced-stage group resided in rural areas (63.63%) compared to the lower-stage group (50%), but this difference was not statistically significant (*p* = 0.177).

The presence of B symptoms was significantly more frequent in the advanced-stage group (90.90%) than in the lower-stage group (69.23%) (*p* = 0.038). Elevated LDH levels were significantly more prevalent in the advanced-stage group (93.93%) compared to the lower-stage group (57.69%) (*p* = 0.013). Regarding comorbidities, no significant differences were found between the two groups for cardiovascular diseases, obesity, alcohol consumption, smoking, and diabetes. Similarly, no differences were observed for neurological and thyroid conditions, psychiatric disorders, or previous malignancies (all *p* > 0.05). The only comorbidity that showed a statistically significant difference between the groups was viral hepatic B and/or C infection (*p* = 0.044). These viral chronic infections were grouped together in the statistical analysis.

The distribution of histological subtypes did not differ significantly between the two groups (*p* = 0.961), with DLBCL being the predominant subtype in both. The distribution of IPI scores differed between groups, with a higher percentage of ‘High-Intermediate’ and ‘High’ scores in the late-stage group (totaling 69.69%), compared to the lower-stage group (totaling 49.99%). Although a trend toward higher IPI scores in the late-stage group could be observed, this finding did not reach statistical significance (*p* = 0.068).

Regarding treatment and outcomes, a similar percentage of both R-CHOP and Obinutuzumab-CHOP regimens was administered in both groups. Interestingly, out of the 13 patients with follicular lymphoma who received the Obinutuzumab-CHOP regimen, only one patient from the advanced-stage group experienced relapse during follow-up. Due to the very small sample size, comparative statistical tests were not performed. Only five patients with lower-stage disease benefitted from total surgical excision. Among the surviving patients, the rate of relapse during follow-up was not significantly different between the lower-stage group (28%) and the advanced-stage group (44.82%) (*p* = 0.141).

Table 2 summarizes the demographic, clinical, comorbidity, histological, treatment, and outcome data for the lower-stage and advanced-stage groups, along with the results of statistical comparisons.

### 3.4. Multivariate Analysis of Factors Associated with Relapse

To explore factors influencing relapse among the patients who survived the 60-month follow-up, we conducted a logistic regression analysis, adjusting for potential confounders. Of these patients, 20 (37%) experienced relapse (7 out of 25 in the lower-stage group, and 13 out of 29 in the advanced-stage group). Based on clinical relevance, four variables were included in the logistic regression model: disease stage (advanced, *n* = 29 vs. limited, *n* = 25); viral hepatitis, present in 14 patients (4 limited, 10 advanced), treatment type (chemotherapy plus radiotherapy, *n* = 14 vs. chemotherapy alone, *n* = 40), and International Prognostic Index (IPI) score (high-intermediate or high, *n* = 36 vs. low or low-intermediate, *n* = 18). These were chosen for their prominence in our cohort—stage as our primary comparator, hepatitis for its regional relevance, treatment for its significant stage difference, and IPI for its prognostic value—while keeping the model stable with 20 relapse events. IPI scores were dichotomized into high-intermediate/high vs. low-low-intermediate to balance statistical power and clinical interpretability.

Combined chemo-radiotherapy was associated with a reduced relapse risk compared to chemotherapy alone (OR = 0.28, 95% CI 0.08–0.99, *p* = 0.049), reflecting its more frequent use in lower-stage patients (10/26 vs. 4/33). The advanced-stage disease showed a trend toward higher relapse risk (OR = 2.19, 95% CI 0.71–6.78, *p* = 0.175), consistent with the higher relapse rate observed (44.8% vs. 28.0%). Viral hepatitis B/C also suggested an increased risk (OR = 2.65, 95% CI 0.76–9.22, *p* = 0.126), aligning with its prevalence in advanced-stage cases (10/33 vs. 4/26). High-intermediate or high IPI scores similarly trended toward greater relapse risk (OR = 2.45, 95% CI 0.79–7.62, *p* = 0.120), building on its prognostic role. Model fit was acceptable (Hosmer–Lemeshow *p* = 0.584), though our modest sample size (*n* = 54) and limited relapse events (*n* = 20) tempered statistical power. These findings are summarized in Table 3.

## 4. Discussion

This study aimed to evaluate the demographic, clinical characteristics, prognostic factors, and treatment outcomes of patients diagnosed with primary palatine tonsil Non-Hodgkin lymphoma (NHL) at a major tertiary referral hospital in Western and South-Western Romania. By comparing lower-stage (Ann-Arbor stages I-III) and advanced-stage (Ann-Arbor stage IV) disease, we sought to identify significant differences in patient demographics, comorbidities, and survival outcomes over a 60-month follow-up period.

Regarding demographic factors, age and sex did not differ significantly between the two groups in our cohort (*p* = 0.310 and 0.519, respectively). A notable observation was that patients in the advanced-stage group were more likely to reside in rural areas (63.63%) compared to the lower-stage group (50%). This difference, while not statistically significant (*p* = 0.177), may suggest potential disparities in healthcare access, leading to delayed diagnosis and more advanced disease at presentation. These findings are consistent with previous epidemiological studies on NHL, which have reported a median age of diagnosis between 60 and 65 years and no strong sex predilection [15,16,17]. The observed trend of more advanced disease in rural patients is also in line with studies, suggesting that geographic disparities influence cancer diagnosis and treatment outcomes [18]. Our cohort consisted of 59 patients, including 34 (57.62) residing in rural areas from the Western and South-Western regions of Romania. The high percentage of late presenters observed in our study population (55.93%) may be attributed to the lack of screening programs in this region and delayed access to specialized medical care.

B symptoms (systemic symptoms like fever, night sweats, and weight loss) were significantly more frequent in the advanced-stage group (90.90%) compared to the lower-stage group (69.23%) (*p* = 0.038). Likewise, elevated initial lactate dehydrogenase (LDH) levels were more prevalent in advanced-stage cases (93.93% vs. 57.69%, *p* = 0.013), both serving as indicators of more aggressive disease progression. These findings align with the existing literature, which emphasizes that B symptoms and elevated LDH levels are key prognostic markers in NHL [19,20,21,22]. The International Prognostic Index (IPI) is a well-established tool for predicting outcomes in aggressive non-Hodgkin lymphomas, incorporating factors such as age, disease stage, serum lactate dehydrogenase levels, performance status, and the number of extranodal sites involved [23]. Higher IPI scores have been consistently associated with poorer prognosis and lower survival rates [24]. In our study, a trend for higher IPI scores was observed in the late-stage group, and the *p*-value was close to reaching statistical significance (*p* = 0.068). The relatively small sample size of our study might have limited the interpretation of this result.

One of the most striking findings in our study was the higher prevalence of hepatitis B and/or C infections in the advanced-stage group (30.30%) compared to the lower-stage group (15.38%), with a univariate *p*-value of 0.044 that weakened in multivariate analysis (*p* = 0.126). In Western Romania, general population prevalence is estimated at approximately 4–5% for HBV and HCV, with rural areas slightly higher due to limited screening [25]. Our cohort’s 23.7% rate (14/59) exceeds this background, though without a matched control group, we cannot firmly attribute causality to NHL development. Instead, HBV/HCV may influence morbidity and relapse risk, as suggested by our multivariate trend (OR = 2.65, *p* = 0.126). This finding aligns with previous research, which reported that HBV and HCV infections increase the risk of NHL, particularly diffuse large B-cell lymphoma (DLBCL), by two to three times [26,27,28,29,30]. Hepatitis viruses are believed to contribute to lymphomagenesis through chronic antigenic stimulation, persistent inflammation, and genetic instability. A review from Marcucci et al. (2011) found that patients with hepatitis-associated NHL tend to present with more aggressive disease, supporting our observation of a correlation between hepatitis infection and late-stage presentation [31].

Chemotherapy was the primary treatment modality for both groups, with R-CHOP (Rituximab, Cyclophosphamide, Doxorubicin, Vincristine, and Prednisone or Prednisolone) being the most commonly administered regimen. The Obinutuzumab-CHOP chemotherapy regimen was administered to patients presenting follicular lymphoma, with favorable outcomes (only one of the 13 patients who received this regimen experienced relapse), in accordance with previous studies that demonstrated good outcomes in this particular histological subtype [32,33,34]. Interestingly, a significantly higher proportion of patients in the lower-stage group received combined radiotherapy and chemotherapy (38.46%) compared to only 12.12% in the advanced-stage group (*p* = 0.019). This is because radiotherapy in the late-stage group was primarily used for palliative purposes, reflecting disease severity. Previous studies report that combined chemo-radiotherapy is associated with improved outcomes in lower-stage NHL [35,36]. Total surgical excision could be performed only for the patients who presented with stage I or II disease.

Regarding term outcomes, relapse rates were higher in the advanced-stage group (44.82%) compared to the lower-stage group (28%), though this difference was not statistically significant (*p* = 0.141). Four patients in the advanced-stage group and one patient in the lower-stage group died during the 60-month follow-up period, with all non-survivors having multiple comorbidities and a mean age of 70.2 years. These findings highlight the impact of both disease stage and overall patient health on survival outcomes.

Our multivariate analysis further highlighted the protective role of combined chemo-radiotherapy, which reduced relapse risk by nearly three-quarters (OR = 0.28, *p* = 0.049) among survivors, a finding that echoes its established benefit in lower-stage NHL [34,35]. This aligns with studies showing improved local control with radiotherapy in lower-stage disease, though our data extend this to relapse prevention across stages. The trends for advanced-stage disease (OR = 2.19, *p* = 0.175) and viral hepatitis B/C (OR = 2.65, *p* = 0.126) suggest potential drivers of recurrence, particularly in Romania’s hepatitis-prevalent setting, warranting further exploration with larger cohorts.

This study has several limitations that should be considered when interpreting the results. Firstly, the retrospective nature of the data collection introduces the potential for information bias and incomplete data, which is inherent to chart review studies. Secondly, the relatively small sample size, particularly within subgroups (e.g., patients receiving specific treatments), limits the statistical power to detect some potentially meaningful associations, especially those related to factors influencing relapse within each stage group. A larger, prospective study would be required to confirm some of the observed trends. Thirdly, as a single-center study conducted at a tertiary referral hospital in Western Romania, the findings may not be generalizable to other populations or healthcare settings with different patient demographics, referral patterns, or treatment protocols. Our cohort included a disproportionately high percentage of patients presenting with advanced-stage disease. While this reflects the reality of healthcare access in the region and is a key finding of this study, it does limit our ability to fully characterize the spectrum of palatine tonsil NHL. Finally, while we documented several comorbidities, unmeasured confounding factors, such as detailed socioeconomic status, the precise time to diagnosis from symptom onset, and individual patient health behaviors (beyond alcohol consumption and smoking), could have influenced both stage at presentation and treatment outcomes. Additionally, our multivariate analysis of relapse, while adjusting for key variables, was constrained by the modest sample size (*n* = 54) and limited relapse events (*n* = 20). This restricted us to four predictors to avoid overfitting, yielding wide confidence intervals (e.g., hepatitis OR = 2.65, 95% CI 0.76–9.22) and borderline trends rather than firm conclusions. These potential confounders were not systematically collected in the retrospective chart review.

## 5. Conclusions

This study reveals differences in clinical features and treatment approaches between lower-stage and advanced-stage primary palatine tonsil NHL in Western Romania, though our findings remain preliminary due to the modest sample size. Many patients presented with advanced-stage disease, pointing to possible gaps in early detection in this region that merit further exploration. The higher prevalence of viral hepatitis B and/or C in advanced-stage cases (*p* = 0.044) hints at a possible link to disease severity, but this weakened in multivariate analysis (*p* = 0.126), urging caution and validation in larger cohorts. Treatment patterns varied by stage, with chemo-radiotherapy more common in lower-stage patients and radiotherapy often palliative in advanced cases. Given its limitations, our work adds a regional perspective to extranodal NHL research and suggests early diagnosis and hepatitis screening as areas to investigate further. Prospective, multi-center studies are essential to confirm these observations and guide interventions for palatine tonsil NHL in settings like ours.

## Figures and Tables

**Table 1:** Characteristics, treatment, and outcomes in study population. LDH = lactate dehydrogenase; DLCBL = diffuse large B-cell lymphoma; IPI = International Prognostic Index; RT = radiotherapy.

Characteristic	Lower-Stage N = 26	Advanced-Stage N = 33
Age, mean (SD)	61.34 (10.51)	64.85 (13.36)
Sex, *n* (%)		
Male	12 (46.15)	14 (42.42)
Female	14 (53.84)	19 (57.57)
Area of Residence, *n* (%)		
Rural	13 (50)	21 (63.63)
Urban	13 (50)	12 (36.36)
B Symptoms, *n* (%)	18 (69.23)	30 (90.90)
Initial LDH, *n* (%)		
Normal	11 (42.3)	2 (6.06)
Elevated	15 (57.69)	31 (93.93)
Comorbidities, *n* (%)		
Cardiovascular Disease	14 (53.84)	26 (78.78)
Obesity	12 (46.15)	21 (63.63)
Alcohol Consumption	13 (50)	19 (57.57)
Diabetes	11 (42.3)	18 (54.54)
Smoking	14 (53.84)	16 (48.48)
Viral Hepatitis B and C	4 (15.38)	10 (30.30)
Neurological Conditions	7 (26.92)	9 (27.27)
Thyroid Gland Conditions	5 (19.23)	7 (21.21)
Psychiatric Disorders	1 (3.84)	5 (15.15)
Previous Malignancies	0 (0)	2 (6.06)
Histological Subtype, *n* (%)		
DLBCL	20 (76.92)	26 (78.78)
Follicular Lymphoma	6 (23.07)	7 (21.21)
IPI, *n* (%)		
Low	6 (23.07)	2 (6.06)
Low-Intermediate	7 (26.92)	8 (24.24)
High-Intermediate	10 (38.46)	15 (45.45)
High	3 (11.53)	8 (24.24)
Treatment, *n* (%)		
Chemotherapy	26 (100)	32 (96.96)
o R-CHOP	14 (53.84)	18 (54.54)
o Obinutuzumab-CHOP	6 (23.07)	7 (21.21)
RT and Chemotherapy	10 (38.46)	4 (12.12)
Total Surgical Excision	5 (19.23)	0 (0)
Outcomes, *n* (%)		
Death During 60-month Follow-up	1 (3.84)	4 (12.12)
Surviving patients	25 (96.15)	29 (87.87)
Progression-Free Survival	18 (72)	16 (55.17)
Relapse During Follow-up	7 (28)	13 (44.82)

**Table 2:** Comparison of characteristics between lower-stage and advanced-stage palatine tonsil NHL patients; LDH = lactate dehydrogenase; DLCBL = diffuse large B-cell lymphoma; IPI = International Prognostic Index; RT = radiotherapy.

Characteristic	Lower-Stage	Advanced-Stage	*p*-Value	Statistical Test
Mean Age, years	61.34	64.85	0.310	Mann-Whitney U test
Rural Residence, %	50	63.63	0.177	Fisher’s Exact Test
B Symptoms, %	69.23	90.90	0.038	Fisher’s Exact Test
Elevated LDH, %	57.69	93.93	0.013	Fisher’s Exact Test
Viral Hepatitis B/C Infection, %	15.38	30.30	0.044	Fisher’s Exact Test
DLBCL Subtype, %	76.92	78.78	0.961	Fisher’s Exact Test
High-Intermediate + High IPI Score	69.69	49.99	0.068	Fisher’s Exact Test
RT + Chemotherapy, %	38.46	12.12	0.019	Fisher’s Exact Test
Relapses in Survivors, %	28%	44.82%	0.141	Fisher’s Exact Test

**Table 3:** Multivariate logistic regression analysis of factors associated with relapse in surviving patients.

Variable	Odds Ratio (OR)	95% Confidence Interval (CI)	*p*-Value
Stage (Limited vs. Advanced)	2.19	0.71–6.87	0.175
Viral Hepatitis	2.65	0.76–9.22	0.126
Treatment (Chemo + RT vs. Chemo)	0.28	0.08–0.99	0.049
IPI Score (High-Int/High vs. Low/Low-Int)	2.45	0.63–7.62	0.120

## Data Availability

Data presented in this study are available on request from the corresponding author (data are not publicly available due to privacy and ethical reasons).

## Abbreviations

The following abbreviations are used in this manuscript:

ENT	Ears, Nose, and Throat
NHL	Non-Hodgkin Lymphoma
DLBLC	Diffuse Large B-Cell Lymphoma
IPI	International Prognostic Index
LDH	Lactate Dehydrogenase
RT	Radiotherapy
